# The Effect of Diet on the Survival of Patients with Chronic Kidney Disease

**DOI:** 10.3390/nu9050495

**Published:** 2017-05-13

**Authors:** Jacek Rysz, Beata Franczyk, Aleksandra Ciałkowska-Rysz, Anna Gluba-Brzózka

**Affiliations:** 1 Department of Nephrology, Hypertension and Family Medicine, Medical University of Lodz, Zeromskiego 113, 90-549 Lodz, Poland; jacek.rysz@umed.lodz.pl (J.R.); bfranczyk-skora@wp.pl (B.F.); 2 Palliative Medicine Unit, Chair of Oncology, Medical University of Lodz, Zeromskiego 113, 90-549 Lodz, Poland; olarysz@rmed.pl; 3 Department of Nephrology, Hypertension and Family Medicine, WAM Teaching Hospital of Lodz, Zeromskiego 113, 90-549 Lodz, Poland

**Keywords:** survival, diet, chronic kidney disease, malnutrition, glomerular filtration, mortality

## Abstract

The prevalence of chronic kidney disease (CKD) is high and it is gradually increasing. Individuals with CKD should introduce appropriate measures to hamper the progression of kidney function deterioration as well as prevent the development or progression of CKD-related diseases. A kidney-friendly diet may help to protect kidneys from further damage. Patients with kidney damage should limit the intake of certain foods to reduce the accumulation of unexcreted metabolic products and also to protect against hypertension, proteinuria and other heart and bone health problems. Despite the fact that the influence of certain types of nutrients has been widely studied in relation to kidney function and overall health in CKD patients, there are few studies on the impact of a specific diet on their survival. Animal studies demonstrated prolonged survival of rats with CKD fed with protein-restricted diets. In humans, the results of studies are conflicting. Some of them indicate slowing down of the progression of kidney disease and reduction in proteinuria, but other underline significant worsening of patients’ nutritional state, which can be dangerous. A recent systemic study revealed that a healthy diet comprising many fruits and vegetables, fish, legumes, whole grains, and fibers and also the cutting down on red meat, sodium, and refined sugar intake was associated with lower mortality in people with kidney disease. The aim of this paper is to review the results of studies concerning the impact of diet on the survival of CKD patients.

## 1. Introduction

The prevalence of chronic kidney disease (CKD) is high and it is gradually increasing. Persons with CKD should introduce appropriate measures to hamper the progression of kidney function deterioration as well as to prevent the development or progression of CKD-related diseases. Current guidelines for CKD management recommend dietary and lifestyle modifications, however, they have largely been based on general population studies [1,2]. A kidney-friendly diet may help to protect kidneys from further damage. In early CKD stages the adoption of healthy diet might slow glomerular filtration rate (GFR) decline and decrease the prevalence of complete kidney failure [3]. Patients with kidney damage should limit the intake of certain foods to reduce the accumulation of unexcreted metabolic products but also to protect against hypertension, proteinuria and other heart and bone health problems. Despite the fact that the influence of certain types of nutrients has been widely studied in relation to kidney function and overall health condition of CKD patients, there are few studies on the impact of specific diet on their survival.

## 2. Healthy Lifestyle and Eating Habits

The significance of proper diet in CKD is confirmed in a large retrospective cohort study of Slinin et al. [4] which demonstrated that the mortality rate of predialysis adult patients cared for by a dietitian was 19% lower in comparison to those who did not receive this care. It seems that nutritional treatment in early stages of CKD may prolong life; however, this hypothesis has not been assessed in a prospective randomized clinical trial. The Third National Health and Nutrition Examination Survey (NHANES III) Linked Mortality File assessed the association between four lifestyle factors (diet, physical activity, body mass index—BMI, and smoking) with all-cause mortality among CKD participants [2]. It demonstrated that individuals in the highest quartile of the weighted healthy lifestyle score had a 53% lower risk of death compared with those in the lowest quartile. Abstinence from smoking and regular versus no physical activity were associated with the highest reduction in mortality (46% and 20%, respectively), whereas a BMI of 18.5 to 22 kg/m^2^ was associated with a 30% increased mortality. Healthy diet, including high intake of vegetables, fruits, nuts, whole grains, legumes, and fish and low in saturated fat and sodium was associated with lower rates of age-adjusted all-cause mortality in individuals with CKD. Beneficial effects of diet may be mediated by favorable effects on blood pressure (BP), glucose, and lipids [5,6]. However, this relationship disappeared after adjustment for demographic factors [2]. A plant-based diet was shown to influence survival through its impact on metabolic acidosis and blood pressure [7]. Moreover, such a diet reduced urine parameters of kidney injury [8], decreased the production of potential uremic toxins through the alteration of gut flora [9], diminished body weight, and improved cardiovascular outcomes [7,10]. On the other hand, high consumption of sugary drinks/sodas has been demonstrated to be associated with the incidence of albuminuria, CKD and faster GFR decline in the community [11,12,13]. Moreover, a low-fructose diet was found in a randomized clinical trial to considerably reduce inflammatory biomarkers levels and blood pressure in comparison to a control group consuming a standard amount of fructose [14]. Ricardo et al. [15] revealed that adherence to a healthy lifestyle (including no smoking habits, low BMI, high physical activity and dietary quality) was associated with lower risk of all-cause mortality in people with stage 3 or more advanced CKD. 

## 3. Impact of Malnutrition, Hyperphosphatemia and Salt Intake on Survival

### 3.1. Malnutrition

In CKD patients, protein calorie malnutrition is a frequent condition [16,17]. Also “protein-energy waste” (PEW), defined as abnormally low levels/excessive losses of body protein mass and energy reserves, is commonly observed in this group of patients, especially in those undergoing dialysis [17]. Apart from inappropriate nutrient intake, PEW may be a result of enhanced catabolism due to the presence of oxidative stress, systemic inflammation, abnormal glucose and insulin homeostasis, metabolic acidosis, imbalance in anabolism/catabolism, and vitamin D deficiency [13,18]. Malnutrition and protein-energy wasting has been demonstrated to be strongly related to mortality in CKD patients [19,20], while surrogates of over-nutrition, e.g., obesity or hyperlipidemia improved their survival [21,22]. According to some studies, such “reverse epidemiology” is associated with the fact that the short-term impact of malnutrition and inflammation overwhelms the long-term adverse impact of hypertension, hyperhomocysteinemia, or obesity, meaning that it is more probable that such patient will die from undernutrition than rather than from hypertension or cardiovascular reasons [19,23]. According to a meta-analysis, diet containing 0.6 to 0.8 g protein/kg/day is nutritionally and metabolically optimal for patients in advanced chronic kidney disease [24]. However, dietary protein intake should be increased after the initiation of dialysis. Uribarri J. [25] demonstrated that hemodialysis patients with higher protein intake had improved survival. Also, the post hoc analysis of the Hemodialysis (HEMO) Study found that patients without dietary protein restriction had a better survival than those on a protein-restricted diet [26]. It seems that a short-term intervention can improve short-term survival [19]. Kalantar-Zadeh et al. [20] demonstrated that low serum albumin level and reduced protein level was strongly associated with mortality in hemodialysis patients. They also suggested that the increase in serum albumin over time (preferably to values greater than 3.8 g/dL) could potentially save lives of many dialysis patients [27]. On the other hand, low-protein diet has been found to slow the progression of renal disease, while a normoproteic or high-protein diet may enhance uremic symptoms and hyperphosphatemia [16,28]. Therefore, it seems that a balance should be found. According to recommendations, adequate caloric intake must be ensured and at least 60% of the ingested protein must be of high biologic value or contain a high percentage of essential amino acids to ensure net neutral nitrogen balance [13]. A low-protein diet is recommended for CKD patients since it diminishes uremic symptoms, improves hyperkalemia, hyperphosphatemia, and calcium or sodium balance control [29], protects against oxidative stress which may aggravate progression of CKD [30], and delays the initiation of dialysis [31]. The implementation of low-protein diet should be accompanied by counselling on alternative food choices in order to avoid nutritional deficiencies and appearance of malnutrition [13].

### 3.2. Hyperphosphatemia

Patients with renal impairment progressively lose the ability to excrete phosphorus and therefore, hyperphosphatemia is a common problem in patients with advanced CKD [16] and a strong predictor of mortality in advanced stages [32]. Also physiological adaptations to counteract excessive phosphorus retention cause hyperphosphatemia in CKD stage 4–5 [33]. There are three sources of phosphorus in food. Plant foods contain organic phosphorus (mainly in the form of phytates), animal proteins comprise organic P, while processed food is “enriched” in additives and conservatives containing inorganic phosphorus [32]. Phosphorus in plant foods is of lowest bioavailability (20–40%) due to the fact that humans do not have the degrading enzyme phytase, while inorganic P is the most absorbable (~100%) [25,26]. A small crossover trial (9 participants) of two diets in CKD patients with estimated glomerular filtration rate (eGFR) from 25 to 40 mL/min and normal serum phosphorus underlined the importance of the protein source of phosphate in overall mineral metabolism [34]. In this study, despite equivalent protein and phosphorus concentrations in the diets, participants in a vegetarian diet had lower serum phosphorus levels, a trend toward decreased urine 24-h phosphorus excretion, and significantly diminished fibroblast growth factor 23 (FGF23) levels in comparison to a meat-based diet. Moreover, a meat-based diet was associated with a decrease in parathormon (PTH) concentration.

Excessive consumption of phosphorus-rich food contributes to hyperphosphatemia which can be dealt with via change of diet, the use of phosphorus binders and/or dialysis. A protein-restricted diet was shown to reduce the supply of phosphorus [13]. D’Alessandro et al. [35] designed a phosphorus pyramid which is a simple, visual tool for nutritional education helping to reduce dietary phosphorus intake without affecting adequate protein consumption. This scheme presents foods rich in phosphorus that should be avoided and those preferred with lower phosphorus to protein ratio. According to current recommendations patients with CKD stages 3–5 should reduce phosphorus intake to 800–1000 mg/day, in conjunction with use of phosphate binders if necessary [36]. The analysis of data from the prospective longitudinal Chronic Renal Insufficiency Standards Implementation Study (CRISIS) revealed that higher serum phosphate, even within the normal range, was associated with an increased mortality of patients with CKD stages 3 to 4 [33]. The study of a large veteran population with impaired renal function reported the association between serum phosphate adjusted for creatinine clearance and mortality and an hazard ratio (HR) of 1.2 (1.1,1.4) for each 1 mg/dL increase of serum phosphate in multivariate analysis [37]. In turn, the study by Isakova et al. [38] suggests that dietary phosphate restriction in combination with phosphate binder therapy may play a role in the diminishing of FGF23 levels in patients with CKD stages 3–4 and normal serum phosphate levels. The decrease in FGF23 level, for which elevations seem to be a novel risk factor for end-stage renal disease (ESRD), cardiovascular disease, and mortality, was most pronounced in patients with the highest levels at baseline [38].

Voormolen et al. [39] found the link between of phosphate level and the progression of renal disease and mortality in pre-dialysis patients in the multi-center Primary Prevention Parameters Evaluation (PREPARE) study. Dialysis Outcomes and Practice Patterns Study (DOPPS) demonstrated a better survival and nutritional status in patients using phosphate binders [40]. Other large studies found a relationship between elevated concentration of phosphate and higher mortality of dialysis patients [41,42]. This relationship may be explained by the role of phosphate in the pathogenesis of vascular calcification and left ventricular hypertrophy contributing to cardiovascular disease and death [43,44]. A study by Russo et al. [45] demonstrated a significant reduction in all-cause mortality, dialysis initiation, and composite end-point risk following the use of phosphorus-restricted diet and sevelamer combination (but not a phosphorus-restricted diet alone) in non-dialysis CKD patients with absent or moderate but not accelerated coronary artery calcification (CAC) progression. Therefore, they suggested that phosphorus might have little or no effect on the occurrence and progression of CAC and consequently a phosphorus-restricted diet might not be useful in reducing the vascular calcification process in non-dialyzed CKD patients [45].

Ca × P have been demonstrated to influence cardiovascular outcome and mortality [41,46,47]. Schwarz et al. [48] demonstrated a correlation between higher Ca × P and higher risk of progressive CKD, however it is not known whether it is not phosphorus that predominantly contributed to the increased risk. Moreover, the Dialysis Outcomes and Practice Patterns Study (DOPPS) found greatest risk of mortality associated with high calcium and high phosphorus in dialysis patients [49]. According to Lim et al. [47] high serum phosphorus (>4.2 mg/dL) in combination with low serum calcium (<9.1 mg/dL) accounted for the highest risk of kidney disease progression. It seems that the combination of these two factors is a more accurate clinical marker than serum phosphorus or calcium alone.

### 3.3. Metabolic Acidosis

The frequency of metabolic acidosis increases along with the decrease of renal function, especially when the glomerular filtration rate falls below 30–40 mL/min/1.73 m^2^. The most common negative consequences are as follows: bone demineralization [50], tubulointerstitial fibrosis [51,52], inflammation [53], the stimulation of the renin-angiotensin system [54] and adrenocortiocotrophic hormone [55]. According to studies, metabolic acidosis is also associated with increased cardiovascular risk [56,57,58]. Mechanisms by which metabolic acidosis may stimulate nephropathy progression in CKD involve sustained, high kidney levels of mediators of increased distal nephron acidification in response to GFR reduction, such as endothelin and aldosterone [59]. In CKD patients, low bicarbonate reflects primary metabolic acidosis [60], and it is considered to be a risk factor for mortality and CKD progression [61,62]. Current guidelines recommend treatment with alkali in case of bicarbonate levels below 22 mmol/L in order to prevent the aforementioned complications [63]. The amelioration of metabolic acidosis has been suggested to attenuate CKD progression as well as hard outcomes [17,64]. The correction of metabolic acidosis can be achieved with oral alkali intake in the form of sodium bicarbonate, with a diet rich in fruit and vegetables [65] as well as with very low-protein diet [66]. Kidney Disease Outcomes Quality Initiative (KDOQI) recommends Na^+^ citrate or NaHCO_3_ in the treatment of metabolic acidosis in CKD [67]. However, Na^+^, Na^+^ citrate and NaHCO_3_ may aggravate volume retention and/or hypertension in CKD [66]. 

A case-control study by Di Iorio et al. [66] clearly demonstrated that a very low protein diet (VLPD) containing a high quantity of fruit and vegetables and with a very low amount of protein, supplemented with essential amino acids and ketoanalogs of non-essential amino acids considerably reduced net endogenous acid production (NEAP) by 53% after six months (*p* < 0.0001) and 67% after 12 months (*p* < 0.0001) and potential renal acid load (PRAL) by 120% after six months (*p* < 0.0001) and 138% after 12 months (*p* < 0.0001). Also, the correction of hyperpotassemia as a consequence of a physiological correction of metabolic acidosis was observed after 12 months on the VLPD diet [66]. The consumption of the Dietary Approaches to Stop Hypertension (DASH) diet rich in fruits and vegetables, low-fat dairy, whole grains, lean meats, fish, poultry, nuts, seeds and legumes, with reduced sweets and saturated fat for two weeks was shown to modestly lower serum bicarbonate without causing incident hyperkalemia or resulting in metabolic acidosis onset in patients with eGFR of 30.0–59.9 mL/min/1.73 m^2^ [68]. The results of this small prospective, before–after pilot feeding pilot study suggest that such a diet is beneficial and safe for adults with hypertension and moderate CKD [68]. An interventional, comparative, randomized controlled trial which compared the effect of 36 months of dietary acid reduction with added daily oral NaHCO_3_ (HCO_3_) or added fruit and vegetables (F and V) on eGFR and other kidney parameters supported the thesis that a base-producing F and V diet in individuals with metabolic acidosis and stage 3 CKD (due to hypertensive nephropathy) not only increased plasma TCO_2_, but also reduced urine excretion of angiotensinogen and preserved eGFR [65]. Authors of this study concluded that the inclusion of fruits and vegetables into the baseline diet is kidney protective in individuals with CKD and metabolic acidosis and it is beneficial even for patients with plasma TCO_2_ > 22 mmol/L (a level above which the KDOQI does not recommend treatment [67]). Also in patients with CKD stage 4, one year of dietary acid reduction with base-producing fruits and vegetables or oral NaHCO_3_ resulted in higher baseline plasma TCO_2_ (21.2 ± 1.3 vs. 19.5 ± 1.5 mM; *p* < 0.01 in HCO_3_ group and 19.9 ± 1.7 vs. 19.3 ± 1.9 mM; *p* < 0.01 in fruits and vegetables group), and diminished urine indices of kidney injury in comparison to the state before treatment [69]. Moreover, lower plasma TCO_2_ and higher urine net acid excretion (NAE) were observed in the fruits and vegetables group in comparison to HCO_3_ group, probably due to higher alkali equivalents prescribed to the HCO3 patients than the fruits and vegetables patients [69]. In a quite large prospective Health, Aging, and Body Composition Study [60] involving generally healthy older individuals with a low prevalence of CKD (12%), low bicarbonate was associated with 24% higher risk of all-cause mortality than in persons with normal bicarbonate, and CKD status did not modify this relationship. In a post-hoc analysis of the Modification of Diet in Renal Disease (MDRD) study, patients with stage 2–4 CKD and with low plasma bicarbonate levels had increased risk of adverse outcomes such as renal death and mortality [70]. 

Bellasi et al. [56] study indicated the relationship between metabolic acidosis and insulin resistance in diabetic patients with chronic kidney disease. They also shown that oral sodium bicarbonate administration and/or low protein diet or diet rich in fruit and vegetables prescribed to avoid or correct metabolic acidosis, improved also insulin sensitivity in the CKD + diabetes mellitus (DM) population [56]. 

In light of the aforementioned results, it seems that dietary acid reduction is kidney protective in CKD patients.

### 3.4. Salt Restriction

High dietary sodium is an important factor influencing blood pressure, predisposing patients with established CKD to salt-sensitive hypertension and fluid retention [13]. It also directly causes renal damage. Studies on animal models demonstrated salt-induced kidney damage, indicating that high salt intake invariably aggravates the course of renal damage. Salt-restricted diets were associated with less proteinuria and glomerulosclerosis in unnephrectomized spontaneously hypertensive rats [71]. High sodium intake induces hyperfiltration which, in turn, can cause renal damage as demonstrated in numerous animal studies. Sanders et al. [72] demonstrated that sodium intake decreased (in dose-dependent manner) life expectancy from 26 to 6 months in mice fed with high-sodium diet, which points the detrimental effects of high sodium intake on overall survival.

Lambers Heerspink et al. [73] in their review summarized the plausible mechanisms of impact of salt on renal damage, blood pressure and patient outcomes. Renal damage associated with salt intake may be a result of its interaction with aldosterone. Tertiles of urinary sodium excretion and proteinuria were shown to be higher in subjects with high plasma aldosterone concentrations, which may suggest that high sodium could intensify the risk of progressive renal function loss by mechanisms not related to blood pressure, e.g., pro-fibrotic, effects of aldosterone [74]. Moreover, the Na/K-ATPase inhibitors marinobufagenin and endogenous ouabain were suggested to be potential mediators of salt-induced kidney damage in chronic renal failure. These two inhibitors participate in sodium regulation and also exert pro-fibrotic effects. In a study by Kolmakova et al. [75], the level of plasma marinobufagenin was significantly higher in patients with chronic kidney disease on dialysis in comparison to healthy controls. The effect of high salt intake on GFR was shown in a study comprising healthy persons, in which mean GFR and effective renal plasma flow (ERPF) significantly increased following the change to high sodium intake (both *p* < 0.001) [76]. Also, urinary albumin excretion was increased by high sodium intake from 6.0 (5.4–6.7) to 7.6 (6.9–8.9). In a multicenter, crossover randomized control trial (RTC) involving 52 Dutch outpatients with non-diabetic nephropathy and CrCl ≥ 30 mL/min, dietary sodium restriction to a level recommended in guidelines was more effective than dual renin-angiotensin-aldosterone system (RAAS) blockade for reduction of proteinuria and systolic blood pressure (Slagman et al. [77]). A double-blind, crossover placebo-controlled RCT of 20 hypertensive Australian outpatients with CKD stages 3–4 demonstrated that salt restriction (60–80 mmol daily) might potentially decrease cardiovascular risk and progression of CKD in comparison to the intake of 180–200 mmol daily [78]. In this study, fluid volume, body weight, proteinuria, and albuminuria diminished by 40–50% in the low salt period in comparison to the high salt period. Plasma renin and plasma aldosterone were found to be increased. However, in the small single-center double-blind randomized cross-over trial LowSALT CKD, a significant change was observed in kidney function parameters, with a low sodium diet resulting in a decrease in eGFR of hypertensive stage III–IV CKD patients, reflected by an increase in creatinine and urate in comparison to high sodium diet [79]. Similar results were obtained by Suckling et al. [80] in which a high sodium intake resulted in increased creatinine clearance, at least in the short term in patients with type 1 and type 2 diabetes. This phenomenon could be ascribed to induced hyperfiltration associated with increased intraglomerular pressure [81]. 

## 4. Various Types of Diet

### 4.1. Healthy Dietary Patterns

Recent meta-analysis including seven studies involving 15,285 participants assessed mortality and the risk of progression to ESRD in CKD patients with healthy dietary patterns (rich in fruits, vegetables, fish, cereals, whole grains, and fibers, and deficient in red meat, salt, and refined sugars) and those with less healthy diet [82]. In most of studies (6 out of 7), healthy dietary patterns were consistently associated with lower mortality (adjusted relative risk (RR), 0.73; 95% confidence interval (CI), 0.63 to 0.83). However, no statistically significant association was observed between healthy dietary patterns and the risk of ESRD (adjusted RR, 1.04; 95% CI, 0.68 to 1.40). Therefore, it seems that the implementation of healthy dietary patterns could be an efficient tool to reduce mortality in people with kidney disease [82]. Also a large prospective cohort study [83] of 544,635 community-dwelling adults demonstrated that the highest quintiles of the Alternate Healthy Eating Index (AHEI) (subdistribution hazard ratio—sHR 0.71; 95% confidence interval (CI) 0.65–0.79), Healthy Eating Index (HEI) (sHR 0.82; 95% CI 0.74–0.91), Mediterranean Diet Score (MDS) (sHR 0.84; 95% CI 0.74–0.95), and Dietary Approaches to Stop Hypertension (DASH) (sHR 0.85; 95% CI 0.77–0.94) were associated with a reduced composite risk of major renal outcomes in comparison to the lowest score quintile. Moreover, the highest sodium quintile (sHR 1.17; 95% CI 1.02–1.33 for sodium intake > 3.6 g/day) was shown to have an increase in hazard, whereas the highest potassium quintile (sHR 0.83 (0.73–0.95)) showed a decrease in this hazard [83]. 

A sub-analysis of the large population-based The REasons for Geographic And Racial Differences in Stroke (REGARDS) Study [84] of 3972 black and white US adults aged 45 years or older with CKD (eGFR < 60 mL/min/1.73 m^2^) demonstrated that the consumption of fried foods, organ meats and sweetened beverages (foods common in Southern cuisines) was independently associated with higher risk of mortality. No considerable relationship was found between convenience, sweets/fats or alcohol/salads pattern scores and hazard ratios (HRs) of mortality after the adjustment for age, gender, race, geographic region of residence, energy intake, lifestyle factors, comorbidities, education, income and eGFR. In contrast, consumption of a diet rich in fish, fruits and vegetables was associated with lower mortality risk over time. The highest quartile of plant-based pattern scores was associated with lower risk of mortality in the fully-adjusted model (HR, 0.77; 95% confidence interval (CI), 0.61–0.97) in comparison to the lowest quartile. Also, a graded association between higher Southern pattern scores and higher risk of all-cause mortality was revealed. Participants in the highest quartile had 1.5-fold higher risk of death as compared to participants in the lowest quartile (HR, 1.51; 95% CI, 1.19–1.92) [84].

A prospective population-based cohort Singapore Chinese Health Study which recruited 63,257 Chinese adults assessed the influence of specific types of food on incident ESRD [85]. In this study a strong, positive association between red meat consumption and ESRD risk was observed, even after the adjustment for lifestyle/comorbidity risk factors of ESRD, other food sources of protein and total protein intake (HR comparing the extreme quartiles was 1.38 (95% CI, 1.11 to 1.71; *p*_trend_ < 0.001)). Therefore, increased risk associated with red meat intake (mainly pork—in 97%). was not confounded by higher overall dietary protein intake. Moreover, the probability of ESRD-free survival was higher (but not high) among persons with lower intakes of red meat (quartiles 1 and 2) compared with those with higher intakes (quartiles 3 and 4) (log-rank *p*, 0.001). In this study, no considerable relationships were found between the risk of ESRD and the intake of poultry, fish and shellfish, eggs, and dairy products. Additionally, the replacement of one serving of red meat with one serving of poultry, fish, eggs, or soy and legumes was associated with a significant decline of ESRD risk. The substitution of one daily serving of red meat with poultry resulted in the highest reduction of ESRD risk at 62.4% (95% CI, 33.1 to 78.9; *p*, 0.01), substitution with fish resulted in a 48.6% lower risk of ESRD risk (95% CI, 21.4 to 66.5; *p*, 0.01), while soy and legumes or eggs resulted in a 50.4% (95% CI, 31.3 to 64.2; *p*, 0.01) and 44.9% (95% CI, 13.9 to 64.7; *p*, 0.01) reduction in ESRD risk, respectively [85]. Authors of this study suggested that the observed reduction in risk could be associated with the fact that red meat might produce endogenously more acid that other protein-rich food or with the presence and the role of nitrites, nitrates, heme iron, advanced glycation end-products (AGEs), and advanced lipoxidation endproducts (ALEs) in such meat [85,86].

Finally, numerous studies confirm that the Western diet (also known as meat-sweet diet or standard American diet) characterized by high content of high-fat foods, high-sugar desserts and drinks, red meat and refined grains is not beneficial for health and associated with obesity, cardiovascular complications, colon cancer, osteoporosis and CKD [87,88,89,90]. A subgroup analysis of data from a prospective observational cohort Nurses’ Health Study demonstrated higher risk of microalbuminuria and a rapid decline of estimated glomerular filtration rate in persons consuming two or more servings per week of red meat [91,92]. Western diets were shown to cause the impairment of renal vascular function, inflammation and subsequent microalbuminuria due to the omnipresent sugars which exert negative effects. Brymora et al. [14] demonstrated diminished inflammatory parameters, including high sensitivity C-reactive protein, soluble intercellular adhesion molecule as well as lower fasting serum insulin levels and blood pressure in patients with stage 2 and 3 CKD after they switched to a low-fructose diet for 6 weeks. Moreover, fructose was shown to accelerate metabolic syndrome development and CKD progression [93]. The consumption of two or more high-sugar content beverages has been reported to increase the risk of glomerular filtration impairment and proteinuria [94].

### 4.2. Mediterranean-Like Diet 

The Mediterranean diet, based on the consumption of fruits and vegetables, whole grains, legumes, nuts, olive and canola oil, herbs and spices and limiting red meat, has a low phosphorous content and has been shown to reduce plasma homocysteine, serum phosphorus, microalbuminuria, and cardiovascular risk [95].

Huang et al. [96] study demonstrated that adoption of a Mediterranean-like diet was associated with better kidney function, while low adherence to it resulted in poorer survival rates among individuals with CKD. In this study the Mediterranean Diet Score (MDS) with slight modifications was applied [97,98]. The MDS is a quality score, which uses dietary intake medians as cut-offs for eight food components of a typical Mediterranean diet. Every two-point increase in the MDS was associated with lower total mortality risk (adjusted HR (95% CI) = 0.82 (0.69 to 1.01)). Moreover, CKD individuals with high and medium MDS adherence presented 23% and 25% lower risk of all-cause mortality, respectively (adjusted HR (95% CI) = 0.75 (0.52 to 1.06) and 0.77 (0.44 to 1.36), respectively, *p* for trend = 0.10), compared with low MDS adherence CKD individuals [96]. Again, beneficial effects of the Mediterranean diet were ascribed to alkali-inducing fruits and vegetables which improved metabolic acidosis and attenuated kidney injury [8,69] and to high content of fiber and polyunsaturated fatty acids and low content of saturated fatty acids. Moreover, the Mediterranean diet may deliver relatively low contents of salt and phosphate which positively influence survival in the CKD population [62]. The relationship between low adherence to Mediterranean diet and worse survival among individuals with CKD was confirmed in meta-analysis [99]. 

### 4.3. Moderately Restricted Low-Protein Diets

Piccoli et al. [100] in their study analyzed the impact of moderately restricted low-protein diets on the survival of patients with advanced CKD. The relative mortality risk in the on-diet cohort was significantly lower in comparison with various dialysis populations (United States Renal Data System (USRDS): relative risk—RR: 0.44 (CI 0.36–0.54); Italian Dialysis Registry-RR 0.73 (CI 0.59–0.88) and French Dialysis Registry-RR: 0.70 (CI 0.57–0.85)). A survival advantage was also observed in younger patients (age < 65 years) on dialysis, while mortality rates for older patients were equivalent to those recorded in the two European registries, and lower than those in the USRDS [100]. Their another study revealed that patient survival was higher on a vegan, supplemented diet (low-protein diet supplemented with keto analogues; LPD-KA), while renal survival was better on a diet based on aproteic commercial food (low-protein diet supplemented "aproteic" commercial food; LPD-ACF) [101]. Mortality rate per 100 patient-years of observation was 5.4 per 100 patient-years on the LPD-KA diet and 11.2 per 100 patient-years on the LPD-ACF diet. Moreover, univariate analysis demonstrated that patients on a vegan, supplemented diet had higher risk for starting dialysis in comparison to the second group. However, the impact of diet on mortality and combined outcome disappeared following the adjustment for age and comorbidities (multivariate Cox model). In their study, patients were allowed to consume protein-unrestricted free meals, personalize control schedules, and they did not have to weigh food. Due to the fact that it is difficult to tell a priori which diet is most suitable for a given patient, authors of this study suggested that the LPD-ACF diet may be better for older patients who do not want to change their dietary habits, while LPD-KA may prove more beneficial for younger patients, who can easily fit it in their daily lives [101].

### 4.4. Very-Low-Protein Diet Supplemented with Amino Acids and Ketoacids (s-VLPD)

In animal models, the sVLPD diet has been demonstrated to preserve renal function via the prevention of an adaptive increase in glomerular capillary pressure resulting in progressive glomerular sclerosis, and exerts anti-inflammatory action [102,103,104]. 

For humans, the intake of 0.6 g protein/kg ideal body weight/day was demonstrated to be generally sufficient to maintain body composition in most CKD patients, supposing that proteins are of high biological value (they contain a sufficient amount of essential amino acids to support synthesis of proteins in the body) [105,106]. According to Coresh et al. [107] and Aparicio et al. [102] even a diet comprising 0.3 g protein/kg ideal body weight/day is sufficient provided that a ketoanalog supplement is added to support the synthesis of body proteins and to increase nitrogen intake. Many studies indicate that diminished consumption of animal proteins or vegetarian diet is associated with better outcomes of CKD patients due to the reduced intake of phosphates and salt plus precursors of metabolic acidosis as well as a more beneficial lipid intake [108,109,110]. 

Several studies demonstrated that a very-low protein diet supplemented with amino acids and ketoacids (s-VLPD), providing 0.3–0.5 g protein/kg BW/day, improved several metabolic abnormalities, such as hyperphosphatasemia, metabolic acidosis, hyperparathyroidism and dyslipidemia in non-dialysis patients with moderate-to-advanced chronic kidney disease (CKD) [111,112,113]. Moreover, it was shown to correct proteinuria, blood pressure and hemoglobin without serious adverse effects [114,115,116]. Despite the fact that s-VLPD diet does not slow down the progression of glomerular filtration rate (GFR) worsening, it has been demonstrated to delay renal death, sparing patients with advanced CKD from dialysis by 1–2 years [117,118]. s-VLPD is an option for stage 4–5 CKD patients with the exclusion of individuals with severe comorbid conditions predisposing them to potential hypercatabolic status [102]. The reduction of protein and phosphate intake as a result of the VLPD diet supplemented with ketoanalogs and essential amino acids was shown to decrease indoxyl sulfate (IS) levels in CKD non-dialysis patients [119]. After 1 week of the VLPD diet, a 36% reduction in IS levels was observed in comparison to the LPD diet (7.12 ± 3.89 μM during VLPD vs. 11.1 ± 6.6 μM during LPD, *p* < 0.0001). In CKD patients, indoxyl sulfate is present at high serum levels and it is believed to be involved in glomerular sclerosis, renal interstitial fibrosis, the progression of CKD and in the onset of its complications [119]. Barreto et al. [120] reported that IS serum levels were a powerful predictor of overall and cardiovascular mortality. Moreover, a preliminary study by Di Iorio et al. [121] provided evidence that the VLPD diet, without the use of phosphate binders, was able to reduce FGF23 in stages 3 and 4 CKD patients. Significant diet-associated reduction in P intake obtained with only 1 week of VLPD was sufficient to reduce FGF23 levels. 

A supplemented VLPD diet has also been shown to allow for a long-lasting reduction of erythropoietin (EPO) dose required to maintain hemoglobin level within the recommended range in patients with advanced CKD [122]. Anemia, which poses a common problem in this group of patients, is associated with relative erythropoietin deficiency and disturbed iron homeostasis [123]. Also, the presence of circulating uremic-induced inhibitors of erythropoiesis, shorter lifespan of red blood cells and nutritional and vitamin deficiencies were suggested to be responsible for anemia [124]. VLPD-related reduction in the required EPO dose has been shown to depend on the correction of moderate secondary hyperparathyroidism rather than on the reduction of plasma urea levels [122,125]. Moreover, it was demonstrated that anemia was simultaneously and independently associated with circulating levels of calcium and phosphorus—in patients with advanced non-dialysis CKD the odds of anemia decrease 71% for every 1 mg/dL increase in albumin-corrected serum calcium and increase 119% for every 1 mg/dL increase in serum phosphorus [123]. Also, Tran et al. [126] study confirmed the relationship between serum phosphorus levels ≥ 3.5 mg/dL and both mild and moderate anemia (odds ratio (OR) for moderate anaemia (95% confidence interval): 1.16 (1.04–1.29) for every 0.5 mg/dL phosphorus increase) in persons with early CKD and normal kidney function. Therefore, it seems that very-low-protein diet, which delivers a reduced amount of phosphorus, can diminish the risk of anemia. However, post hoc analysis of the Modification of Diet in Renal Disease (MDRD) study-B provided the controversial hypothesis that that prescription of s-VLPD increases mortality during the subsequent dialysis period [127]. This observation has not been confirmed in other studies. A meta-analysis of five trials including the MDRD study revealed a considerable reduction in the risk of renal failure and deaths (RR = 0.67) over 18–36 months of follow-up [117]. Also, a meta-analysis by Fouque et al. [128] has also demonstrated 39% reduction in the number of renal deaths in patients on LPD or s-VLPD. The randomized controlled Diet or Dialysis in Elderly (DODE) trial, which evaluated the non-inferiority of s-VLPD compared to dialysis on mortality in older patients failed to reveal a survival disadvantage for patients on an s-VLPD diet in comparison with those on dialysis [118]. Also, in younger patients with CKD long-term outcome was not negatively influenced by s-VLPD treatment [129]. A long-term, historical, cohort, controlled study comparing the impact of very-low protein diet supplemented with amino acids and ketoacids used in patients undergoing conservative treatment of CKD and the general CKD population did not increase mortality risk in the subsequent dialysis period. Mortality of patients consuming s-VLPD was 8/100 patients/year, while in tertiary nephrology care (TNC) it was 10/100 patients/year) [111]. Such mortality was lower than in unselected CKD population (CON) (14/100 patients/year). Moreover, the comparison of GFR at the beginning of dialysis therapy in s-VLPD and TNC patients revealed lower GFR values in the first group (5.4 vs. 10.6 mL/min) suggesting that very low-protein diet prolonged dialysis-free period (by at least 1 year) even in the presence of a more severely reduced residual renal function. Despite more advanced CKD and considerably reduced renal function at the start of dialysis, the survival time was the same in s-VLPD and TNC groups [111]. Bellizzi et al. [111] suggested that s-VLPD diet can be used as a part of the intensive care delivered to CKD patients. The introduction of a low-protein diet requires a self-discipline, help of dietitians and a close monitoring by nephrologists.

## 5. Conclusions

Appropriate nutrition is vital for patients with CKD at all stages. Numerous studies indicate that diet rich in fruits, vegetables, fish, cereals, whole grains, fibers and polyunsaturated fatty acids but low in saturated fatty acids is beneficial for CKD patients. Also, a very-low protein diet supplemented with amino acids and ketoacids (s-VLPD) was found to be safe and advantageous for patients with chronic kidney disease, especially at stages 4–5, due to the fact that it corrects proteinuria, blood pressure and hemoglobin and therefore it may prolong not only the dialysis-free period but also the survival. However, switching diets should be carefully consulted with a nephrologist and monitored by a dietician to avoid nutritional errors which can accelerate kidney function deterioration.
